# The methanolic extract of Garcinia atroviridis (MeGa) reduces body weight and food intake, and improves lipid profiles by altering the lipid metabolism: a rat model

**DOI:** 10.3906/biy-2005-2

**Published:** 2020-12-14

**Authors:** Wai Feng LIM, Suriati Mohd NASIR, Lay Kek TEH, Richard Johari JAMES, Mohd Hafidz Mohd IZHAR, Mohd Zaki SALLEH

**Affiliations:** 1 Integrative Pharmacogenomic Institute (iPROMISE), Universiti Teknologi MARA Selangor, Selangor Darul Ehsan Malaysia; 2 Faculty of Pharmacy, Universiti Teknologi MARA Selangor, Selangor Darul Ehsan Malaysia; 3 Comparative Medicine and Technology Unit, Institute of Bioscience, Universiti Putra Malaysia, Selangor Malaysia

**Keywords:** Obesity, *Garcinia atroviridis*, phytochemistry, toxicity, antiobesity, metabolomics

## Abstract

*Garcinia* species are widely used for their slimming effects via increased fat burning and suppression of satiety. However, scientific evidence for the biological effects of *Garcinia atroviridis* (GA) is lacking. We investigated the phytochemical composition, safety profiles, and antioxidant and antiobesity effects of methanolic extracts of *Garcinia atroviridis* (MeGa) in obese female rats. Repeated dose toxicity studies were conducted according to the OECD guidelines. Upon sacrifice, haematological, biochemical, lipid profile, and serum-based metabolomics analyses were performed to evaluate metabolic expression changes and their related pathways. MeGa contains several phytochemical groups and GA fruit acids. MeGa was found to be nontoxic in both male and female rats with an oral lethal dose (LD50) of 2000 mg/kg. After 9 weeks of treatment, MeGa-treated obese rats had lower weight gain and better lipid profiles (cholesterol and triglyceride), which correlated with the altered metabolic pathways involved in the metabolism of lipid (glycerophospholipid) and biosynthesis of unsaturated fatty acid. In addition, MeGa caused differential metabolism pathways of arachidonic acid and tryptophan that affect the inflammatory response and suppression of appetite. We concluded that MeGa is safe, and its slimming effects are due to the differential metabolism of lipids.

## 1. Introduction

More than 650 million people were obese in 2016, as reported by the World Health Organization (WHO)World Health Organization (2020). Fact Sheet. Obesity and overweight [online]. Website https://www.who.int/en/news-room/fact-sheets/detail/obesity-and-overweight [accessed 2 April 2020].; the numbers were increasing at an alarming rate in 2018. Health professionals such as doctors, pharmacists, and nurses have important roles in advising patients and the general public on weight management strategies, which include appropriate diet with the correct nutrients. Diet is, therefore, the indispensable factor that influences the ability to lose weight or maintain body weight (Keränen et al., 2009; Soeliman and Azadbakht, 2014). Therefore, evidence-based knowledge on local fruits with beneficial effects on weight management may provide practitioners with safer alternatives for developing weight management strategies in their patients as well as the general public.

‘AsamGelugor’ or ‘Asamkeping’ is the popular name for *Garcinia atroviridis* (GA)Griff. ex T. Anders in Malaysia (Alsarhan et al., 2014). It is widely distributed in Peninsular Malaysia, Thailand, Myanmar, and India (Mackeen et al., 2000; Hamidon et al., 2017). Almost all parts of the tree such as fruit, leaves, roots, and stem bark can be used either for food seasoning or medicinal purposes, including antioxidant, antiobesity, antiinflammatory, and cytotoxic activities (Pangsuban et al., 2009; Hamidon et al, 2017). GA leaf extract possesses antioxidant properties with proton-donating ability serving as a free-radical scavenging agent (Nursakinah et al., 2012). The presence of hydroxycitric acid (HCA) in GA has been documented to have potent antioxidant potential (Rittirut and Siripatana, 2006). HCA originated from *Garcinia* species has been widely used around the world as a food supplement for its claimed benefit in weight management for decades. HCA is able to control body weight and appetite by blocking the lipogenesis process (a process that converts carbohydrates to fat) through the inhibition of ATP-citratelyase production in cells (Chuah et al., 2012; Hamidon et al., 2017). Furthermore, HCA is believed to improve the level of blood cholesterol and dilation of blood vessels, and reduces excessive fat absorption (Yapwattanaphun et al., 2002).

Overweight and obesity are measured with body mass index (BMI) and are characterized by an abnormal or excessive deposition of fat tissues when energy intake and expenditure are imbalanced, attributed mainly to poor dietary choices (imbalanced and high fat diet) and physical inactivity (sedentary behaviour) (WHO, 2020). It exerts profound perturbation on the metabolism, mainly in lipid metabolism and glucose metabolism, which may lead to hyperlipidemia, hypertension, and diabetes (WHO, 2020). Obesity-associated metabolic changes can be tracked through altered metabolites (small molecules) in a cell, tissue, or organism. For example, altered metabolites associated with obesity phenotypes have been identified and are linked to BMI changes in humans (Abu Bakar et al., 2015; Park et al., 2015; Cirulli et al., 2019). The metabolic phenotypes can be interpreted biochemically and biologically using these identified metabolites through metabolomics study (identifying all metabolites in a biological sample). It is useful to identify the biomarkers and metabolic pathways associated with obesity in designing and developing better therapeutic targets. Therefore, the metabolomics approach was used to identify the obesity-affected mechanisms underlying the antiobesity activity of MeGa extract in a rat model.

Although herbs are derived from the nature, their safety is not guaranteed, and some may have unexpected adverse effects, including acute liver injury and fatal herb–drug interaction (Crescioliet al., 2018; Kothadia et al., 2018). Many herbal products lack scientific evidence on their safety profiles and their respective mechanisms of actions before they are marketed. Despite many studies reporting on the antioxidant and weight reduction properties of the leaves and fruit extracts of GA (Alias et al., 2017; Hamidon et al., 2017; Lumbantobing et al., 2017), no comprehensive study is available on the safety of GA extracts according to standard guidelines such as good laboratory practice and OECD. Therefore, phytochemical composition and safety limits of the methanolic extract of GA (MeGa) fruit were investigated and a metabolomics study was further conducted to study the changes of metabolic pathways in obese rats treated with MeGa.

## 2. Materials and methods

### 2.1. Chemicals and reagents

Methanol (Merck, Darmstadt, Germany), formalin, and diethyl ether of analytical grade were obtained from Fisher Scientific (Bridgewater, NJ, USA). Other chemicals used in this study include magnesium, hydrochloric acid, concentrated sulphuric acid, 10% ferric chloride, chloroform, stable free radical 2,2-diphenyl-1-picrylhydrazyl (DPPH), Trolox, Folin–Ciocalteu reagent, sodium carbonate, gallic acid, ethanol solution, and 2% (w/v) aluminium chloride.

### 2.2. Analysis of the methanolic extract of Garcinia atroviridis (MeGa)

#### 2.2.1. Preparation of the MeGA

*Garcinia atroviridis* (GA) fruits were collected from a local village in Seremban, Malaysia. The plant specimen was sent for species identification to the Forest Research Institute Malaysia (FRIM); a certificate with sample no. PID 110214-07 was obtained. Ripe fruits were plucked and chopped into small pieces and dried using a FreeZone Plus 12 Liter Cascade Freeze Dryer (Labconco, Kansas City, MO, USA) before being ground by mechanical grinder (SY-04, Himitzu, Japan). The powdered samples were stored in an air-tight bottle. The powdered samples (100 g) were soaked in methanol (ratio of powder to solvent: 1:20) for 3 days before filtering using Whatman No 1 filter paper (Whatman™, Dassel, Germany). The filtrate was concentrated using a Rotavapor® R-210 Rotary Evaporator System (Buchi, Flawil, Switzerland) at a setting of not more than 50 °C. Pure methanolic extract of GA (MeGa) was stored at 4 °C until further analysis. The yield of the extract was calculated as percentage yield (%) = {[Weight of dry crude extract (g)/weight of plant material before extraction (g)] ×100%}.

#### 2.2.2. Phytochemical analysis and assessment of antioxidant assay

Various phytochemical groups (flavonoid, phenol, tannin, saponin, steroid, phlobatanin, and terpenoid) were identified using methods described by Harborne (1998) and Kokate (2005). Total flavonoid content (TFC) and total phenolic content (TPC) of MeGa were quantitated spectrophotometrically with the aluminium calorimetric method (quercetin as the reference standard) and Folin–Ciocalteu method (gallic acid as the reference standard), respectively. The antioxidant capacity of MeGa was assessed using 2,2-diphenyl-1-picrylhydrazyl (DPPH) free radical-scavenging assay (Trolox as the reference standard) with slight modifications (Yang et al., 2011). All of the experiments were conducted in triplicate following the protocol previously reported by Abdul Hisam et al. (2018).

#### 2.2.3. Metabolite profiling of the MeGa

Liquid chromatography mass spectrometry/quadrupole time of flight (LC/MSQ-TOF) was used to profile compounds in MeGa. The stock solution (1 mg/mL) was first purified using the solid phase extraction method (Bond Elut C18, Agilent Technologies, Santa Clara, CA, USA) before being analysed by LC/MSQ-TOF (model 6520 Agilent Technologies). A ZORBAX Eclipse Plus C18 column set (100 mm × 2.1 mm × 1.8 µm; Agilent Technologies) at 40 °C was used. The mobile phases consisted of water with 0.1% formic acid (A) and acetonitrile with 0.1% formic acid (B). The flow rate was set at 0.25 mL/min with 5% of mobile phase Band 95% of mobile phase A over a linear gradient of 36 min. The total run time was 48 min per sample analysis. The electrospray ionization (ESI) source (capillary voltage 4000 V, skimmer 65 V, and fragmentor 125 V), the nebulizer (45 psi), the nitrogen drying gas (flow rate at12 L/min, 350 °C) were set up properly. Data acquisition was collected from a full scan over 100 to 1000 *m/z* in positive ESI mode. During the analysis, both reference masses of C5H4N4 (121.0509 *m/z* ) and C18H18O6N3P3F24 (922.0098 *m/z* ) were injected continuously to deliver accurate mass signals.

### 2.3. Animal experiments and dietary treatments

Sprague-Dawley rats were donated by the Laboratory Animal Facility and Management (LAFAM), Faculty of Pharmacy, Universiti Teknologi MARA (UiTM). Rats were randomly assigned according to the study protocol. Rats (180–220 g) were given 7 days to acclimate to the new laboratory conditions prior to dosing. The rats were individually housed in a ventilated caging system and exposed to a regular 12-h light–dark cycle at room temperature set at 27 ± 2 °C. The rats were allowed to access the food and water ad libitum. Dietary descriptions are detailed in Table A1. Briefly, the rats were fed with normal fat diet (NFD) (D12450B, Research Diets Inc., New Brunswick, NJ, USA) for the control/lean group and high fat diet (HFD) (D12492, Research Diets Inc.) for the obese group. Rats were euthanized by cervical dislocation at the defined time after collecting blood samples as necessary. The research proposal was approved by the local research ethics committee on animal study (UiTM Care, Ref: 69/2015).

### 2.4. Rat model for toxicity study

#### 2.4.1. Acute toxicity effect of the MeGa

An acute toxicity study was performed according to the stepdown procedure in accordance with the OECD 423 guidelines. This is a short-term toxicity study to determine the dosage of MeGa for a subsequent subacute toxicity study. For control (n=3 female rats) and test (n=3 female rats) groups, Sprague-Dawley rats were orally administered distilled water and 2000 mg/kg of MeGa, respectively. Clinical signs of toxicity in the rats were observed for the first 30 min, then 4 h, and daily thereafter up to 14 days. The mortality rate was recorded.

#### 2.4.2. Subacute toxicity effect of the MeGa

According to the OECD 407 guideline, the subacute toxicity study of MeGa extract was conducted using a repeated dose over 28 days. Body weights of 4-week-old Sprague-Dawley rats were recorded and randomly assigned to control, test, and satellite groups (10 rats/group) according to sex. The limiting dose (1000 mg/kg) was prepared in distilled water and orally administered as a single daily dose to the test group and satellite group, while the control group received only distilled water orally. Toxic manifestations and mortality were monitored daily. Body weights, food intake, and water intake of all of the rats were recorded daily. The rats were euthanized after 28 days of treatment except for the satellite group, the rats of which were observed for another 14 days without treatment.

#### 2.4.3. Haematological and biochemical analysis

Blood samples were withdrawn via cardiac puncture for the haematological and biochemical analyses using EDTA-anticoagulated whole blood and serum from clotted blood, respectively. Haematological analysis was performed using a fully automated haematological analyzer (Advia 2120i Siemens, Washington, DC, USA) at the Centre of Pathology Diagnostic and Research Laboratory (CPDRL), Universiti Teknologi MARA, Sungai Buloh. Biochemical analysis was performed at the Veterinary Laboratory Service Unit (VLSU), Universiti Putra Malaysia (UPM).

#### 2.4.4. Relative organ weight

Rats were euthanized by cervical dislocation on the 29th day. Brains, hearts, lungs, livers, kidneys, and spleens were excised, weighed, and examined for any macroscopic changes. The relative organ weights were calculated as relative organ weight (%) = {[Absolute organ weight (g)/body weight of rats on sacrifice day (g)] × 100%}.

#### 2.4.5. Histopathological examination

For histopathological examination, vital organs such as kidneys and livers were preserved in 10% formalin before sectioning. Thin tissue sections were stained with haematoxylin–eosin (H&E) stain and examined for any morphological changes and abnormalities under a light microscope at VLSU, UPM.

### 2.5. Rat model for antiobesity study

Female 4-week-old Sprague-Dawley rats were induced to be obese using a high-fat diet (HFD). After 6 months of feeding, the rats achieved an average of more than 10% increase in weight compared to the lean group; they were classified as moderately obese according to a previous definition (Thibault, 2013).

#### 2.5.1. Antiobesity effect of the MeGa

The rats were randomly grouped (n=6 rats/group) to receive treatment as follows: (i) lean rats without treatment (negative control); (ii) moderately obese rats without treatment (positive control); (iii) moderately obese rats treated with 15 mg/kg of Adipex (positive control with treatment); (iv) moderately obese rats treated with 100 mg/kg of MeGa (Ga-1); (v) moderately obese rats treated with 200 mg/kg of MeGa (Ga-2); (vi) moderately obese rats treated with 400 mg/kg of MeGa (Ga-3). Body weights of the rats were recorded weekly.

#### 2.5.2. Biochemical analysis of antiobesity rat model

Blood samples were withdrawn from the orbital sinus of the rats at 2 different time points, i.e. pretreatment and posttreatment. Serum was then used for the measurement of lipid profiles.

#### 2.5.3. Metabolite profiling of antiobesity rat model

A total of 200 µL of serum was diluted with 150 µL of dH20 (pH10); 300 µL of cold acetonitrile was added, followed by vortex mixing for 1 min for protein precipitation. The samples were centrifuged for 10 min at a speed of 10,621 *g* (Eppendorf Centrifuge 5804R; Eppendorf North America, Framingham, MA, USA), and the supernatant was subjected to protein precipitation another 2 times. The total supernatant collected was spin-dried using a centrifugal concentrator (Eppendorf Concentrator Plus). Dried sample was then reconstituted with 40 µL of mobile phase (water:acetonitrile, 95:5) followed by a brief vortex mixing of 1 min, and then spun at 10,621 *g* for 10 min. Thirty (30) µL of the supernatant was transferred into an autoinjector vial and injected into the LC/MSQ-TOF system for analysis.

#### 2.5.4. Compound identification

The LC/MS QTOF output was analysed using Mass Profiler Professional Version B.12.01 (Agilent Technologies), which provided multivariate statistical analysis for comparative metabolite profiles. Principal component analysis (PCA) was used to examine the clusters and outliers from the input data sets without prior information on sample grouping. A3-dimensional data set of the aligned features detected in all of the biological replicates was generated for visualisation. One-way analysis of variance (ANOVA) determined changes in the metabolites across groups after Benjamin Horchberg correction. Identified metabolites were annotated by mass analysis using the METLIN databasehttps://metlin.scripps.edu/landing_page.php?pgcontent=mainPage and Kyoto Encyclopedia of Genes and Genomes (KEGG) databasehttp://www.genome.jp/kegg/ before pathway analysis was performed using Metaboanalysthttps://www.metaboanalyst.ca/.

### 2.6. Statistical analyses

Statistical analyses for haematological and biochemical parameters were carried out using the SPSS, version 20. The data were expressed as the mean ± standard error of the mean (SEM) values. An independent t-test was used to compare the mean of 2 groups. To compare among groups, one-way analysis of variance (ANOVA) was used followed by a post hoc test, Tukey LSD, to identify the paired data that differed significantly. To compare pre- and posttreatment, the paired sample t-test was used. Data was considered statistically significant when P-value was less than 0.05.

## 3. Results

### 3.1. Phytochemical analysis, antioxidant activity, and metabolite profiling of the MeGa extract

The yield of the methanolic extract of *Garcinia atroviridis* (MeGa) was approximately 9%–10% after complete drying. Phytochemical screening of MeGa revealed the presence of flavonoid, phenol, saponin, and terpenoids; tannin, steroid,and phlobatanin were not detected. The total phenolic and flavonoid contents of MeGa were 8.833 ± 1.18 μg/mg gallic and 0.821 ± 0.08 μg/mg quercetin, respectively. For DPPH antioxidant ability, the IC50 of MeGa (173.33 ± 13.33 μg/mg) was higher than Trolox (IC50 of 54.67 ± 2.67 μg/mg), suggesting that MeGa has a statistically lower radical scavenging ability than Trolox. The chromatographic profiles of the extracts were obtained from different batches of MeGa extracts. There are 5 metabolites that were consistently present in all the batches of MeGa extracts: hydroxycitric acid, 2-(butoxycarbonylmethyl)-3-butoxycarbonyl-2-hydroxy-3-propanolide, tartaric acid, malic acid, and ascorbic acid (Table A2).

### 3.2. Toxicity studies

#### 3.2.1. Acute toxicity study of the MeGa

No visible toxicity sign was observed in the rats treated with 2000 mg/kg of MeGa. Neither behavioural changes nor mortality were observed in the rats over 14 days. The LD50 (lethal dose, 50%) of the MeGa was higher than 2000 mg/kg, as no lethal effect was recorded. According to the Globally Harmonized System of Classification and Labelling of Chemicals (GSH), MeGa is classified under Category 5, as it is considered safe with low toxicity effect. Therefore, 100 mg/kg, 200 mg/kg, and 400 mg/kg oral doses of MeGa were applied in the subsequent subacute toxicity study.

#### 3.2.2. Subacute toxicity study of the MeGa

There were no observable behavioural changes in the MeGa-treated group versus the untreated group. Body weight, food intake, haematological indices, biochemical parameters, and relative organ weights (Table A3) were compared between the MeGa-treated and untreated rats. There were no deaths recorded in the rats of either sex from the treatment groups or the control group. MeGa-treated and control rats appeared to be healthy throughout the 28 days of the treatment period. Subacute toxicity results were comparable to the earlier acute toxicity study on MeGa.

#### 3.2.3. No histopathological changes in liver and kidney tissues

**Figure 1:**
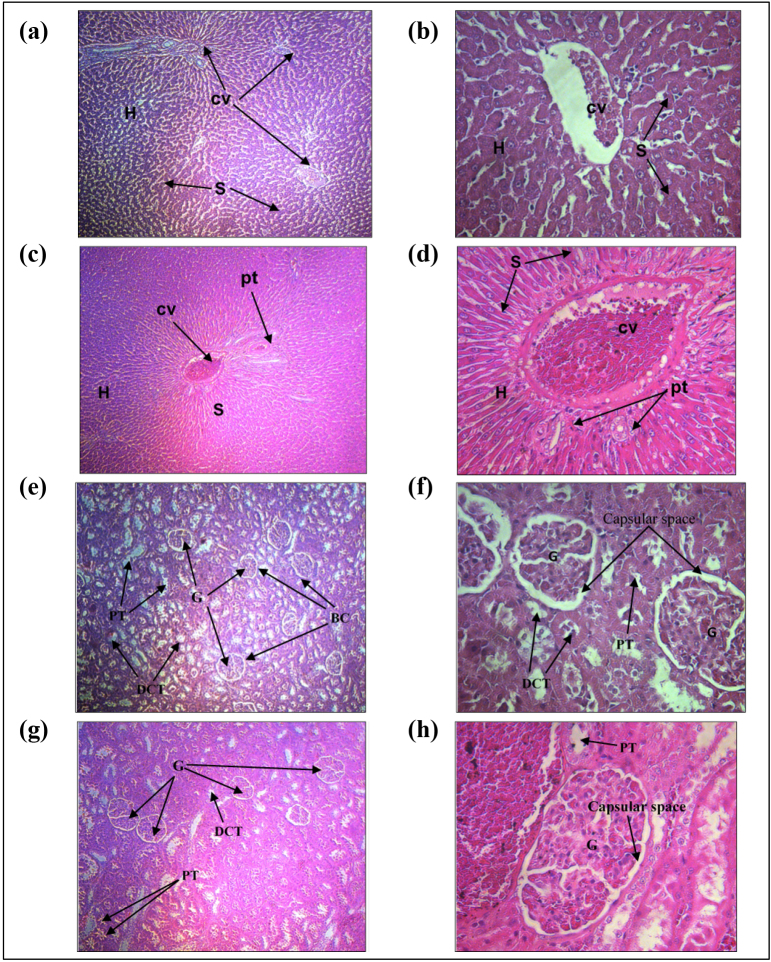
Histopathological examination of liver and kidney cells in the Sprague-Dawley rats for the control and MeGa treated groups. Liver cells of the (a) control rat with H&E stain ×10 magnification, (b) control rat with H&E stain ×40 magnification, (c) treated rat with H&E stain ×10 magnification, (d) treated rat with H&E stain ×40 magnification. Kidney cells of the (e) control rat with H&E stain ×10 magnification, (f) control rat with H&E stain ×40 magnification, (g) treated rat with H&E stain ×10 magnification, (h) treated rat with H&E stain ×40 magnification. Abbreviation: CV = convoluted tubule.

Histopathological analysis was performed on the liver and kidney samples to examine for structural abnormalities on the hepatocytes (liver cells) and kidney cells after treatment with MeGa. The liver samples showed normal architecture with well-defined cytoplasm, cell lining, and round nuclei in hepatic cells (Figures 1a–1d). Histological study of the kidneys for both control and treated groups displayed normal glomeruli networks of small blood vessels surrounded by the Bowman’s capsule and proximal and distal convoluted tubules, without any sign of inflammation (Figures 1e–1h).

### 3.3. Antiobesity study

The rats in whom obesity was induced for 6 weeks using a high-fat diet (HFD) had body weights which were statistically different from rats in the lean group (Figure A1). These obese rats were then treated with MeGa or Adipex.

#### 3.3.1. Reduction in body weight and food intake in MeGa-treated rats

**Figure 2:**
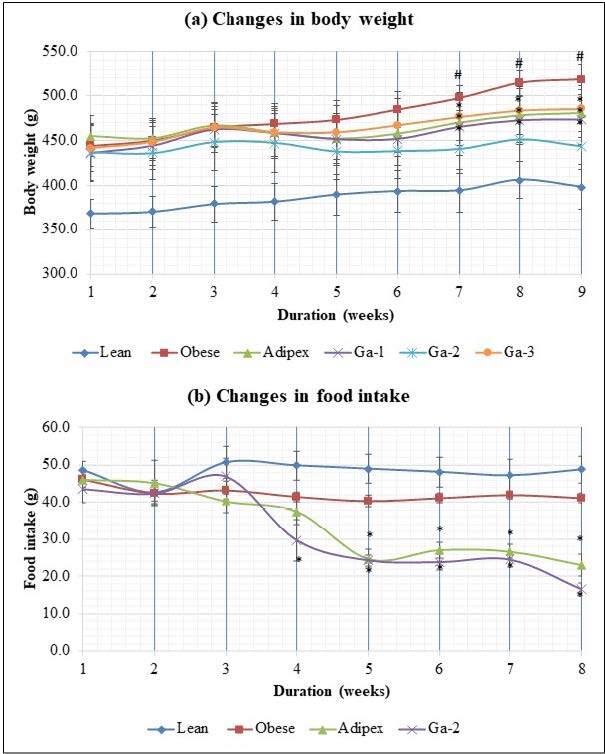
Antiobesity assessment of the MeGa extract in different treatment groups. The changes of (a) body weight (b) food intake in lean (normal control group fed with normal fat diet (NFD) (also called chow diet) that contains 10% fat, obese (experiment group fed with high-fat diet containing 60% fat), Adipex (experiment group fed with high-fat diet and treated with Adipex drug) and Ga [experiment groups fed with MeGa, i.e. 100 mg/kg (Ga-1), 200 mg/kg (Ga-2) and 400 mg/kg (Ga-3)], * indicates significant difference compared with lean group, P-value < 0.05), # indicates significant difference compared to the Ga-2 group, P-value < 0.05) using ANOVA post hoc, Tukey LSD. Body weight gains around 10%–25% compared to lean control were considered moderate obesity.

Obese rats treated with MeGa showed lower body weights than untreated obese rats. The most effective dose for weight reduction was seen in the Ga-2 group (200 mg/kg MeGa), whereby their body weights showed no significant differences from those of the lean group (Figure 2a). After 4 weeks of treatment, the amount of food consumed by the rats treated with Adipex and the Ga-2 group was reduced compared to the obese and lean rats. Lean and obese groups showed similar food intake patterns, while both Adipex and Ga-2 groups had reduced food intakes. These changes were noticeable after 3 weeks of treatment (Figure 2b).

#### 3.3.2. Improved lipid profiles in MeGa-treated rats

**Figure 3:**
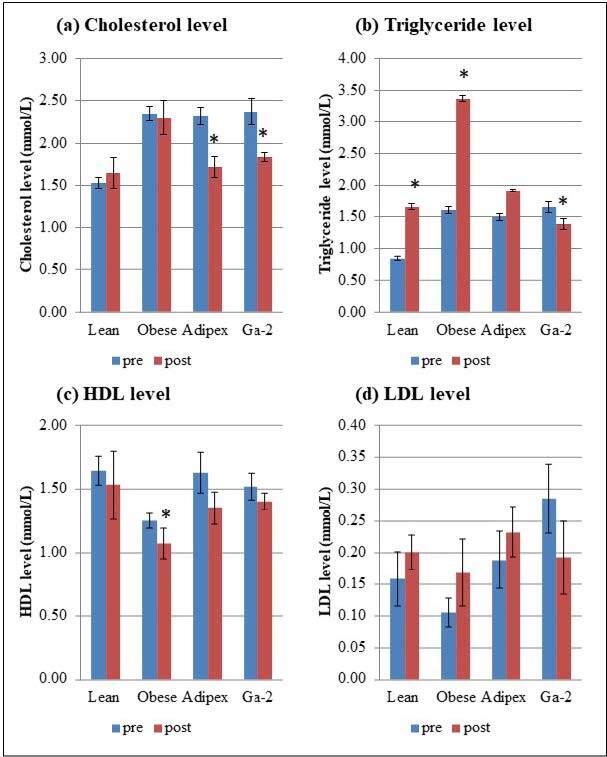
Lipid profile of rats in lean, obese, Adipex- and Ga-2 treated groups. (a) Cholesterol level, (b) triglyceride level, (c) high-density lipoprotein (HDL) level and (d) low-density lipoprotein (LDL) level. * indicates significantly different pretreatment group, P-value < 0.05 using paired sample t-test.

The obese group fed with a high fat diet (HFD) was found to have increased levels of total cholesterol, triglycerides, and LDL (P-value < 0.05), and a decreased level of HDL compared to the lean rats. In the Adipex group, the cholesterol level measured posttreatment was statistically significantly reduced compared to the pretreatment cholesterol level. The posttreatment HDL level was slightly reduced compared to pretreatment, while triglyceride and LDL levels were slightly increased (without a statistical difference) in the Adipex-treated rats. In the Ga-2 group, both cholesterol and triglyceride levels were significantly decreased in the posttreatment rats. For HDL and LDL, the posttreatment levels were decreased in the Ga-2–treated rats (Figure 3).

#### 3.3.3. Metabolite profiling of MeGa-treated rats

**Figure 4:**
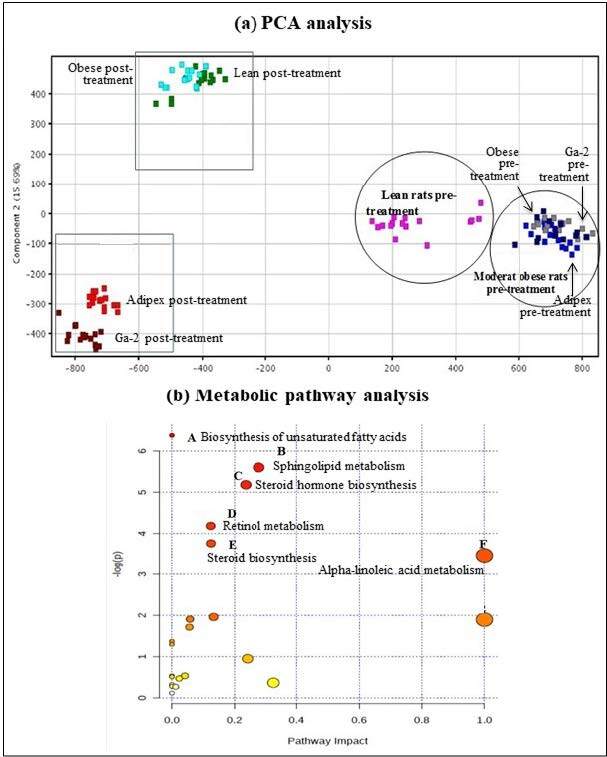
Metabolite profiling of rats in lean, obese, Adipex- and Ga-2 treated groups. (a) PCA score plot of metabolite profiles of rats in different treatment using Mass Profiler Professional software. (b) Metabolic pathway analysis using Metabolomics Pathway Analysis (MetPA) software.

The chromatograms for samples obtained from the lean, obese, Adipex-treated, and MeGa-treated rats demonstrated different metabolite profiles (Figure A2). Principal component analysis (PCA) illustrated distinct separation of the metabolite profiles between the lean rats and obese rats as well as pretreatment and posttreatment (Figure 4a). Metabolic pathways that were discovered to be associated with obesity and in response to MeGa included the alpha-linolenic acid metabolism (impact: 1), sphingolipid metabolism (impact: 0.27821), steroid hormone biosynthesis (impact: 0.23648), tryptophan metabolism (impact: 0.13284), steroid biosynthesis (impact: 0.12448), retinol metabolism (impact: 0.12371), tyrosine metabolism (impact: 0.05788), and the biosynthesis of unsaturated fatty acids (impact: 0) (Figure 4b). The differentially regulated metabolites are shown in Table A4.

## 4. Discussion

As reported in the literature, the methanolic extract of *Garcinia atroviridis* (MeGa) containsflavonoid, phenol, saponin, and terpenoid (Nursakinah et al., 2012). A previous study had shown that the concentration of phenolic compounds is correlated with the antioxidant capacity of an extract (Chatha, 2014). Antioxidant capacity is one of the commonly determined parameters for bioactive components with pharmacological activities. MeGawas tested using DPPH assay for free-radical scavenging ability; its antioxidant activity was significantly lower than that of Trolox. The antioxidant activity exhibited by MeGa indicates its potential to neutralise free radical compounds. *Garcinia atroviridis* (GA) has been reported to have ow antioxidant activity as determined by DPPH assay (Abdullah et al., 2013). The availability and stability of phenolic and flavonoid may contribute to this antioxidant activity. Among the GA fruit acids, hydroxycitric acid (HCA), citric acid, tartaric acid, and ascorbic acid contribute to the antioxidant activity of GA (Rittirut and Siripatana, 2006; Hamidon et al., 2017). HCA was detected as one of the main compounds in MeGa. As reported previously, HCA is believed to induce weight loss via reducing the lipogenesis process and through the suppression of appetite (Chuah et al., 2012).

In this study, rats treated with MeGa had a reduced rate for the increment of body weight and no significant difference in the body weight increment when compared to the lean group. This could be due to the inhibition of fatty acid synthesis by HCA in MeGa. In the liver, HCA acts as a potent competitive reversible inhibitor of ATP-citrate lyase and an inhibitor that disrupts fatty acid synthesis (Chuah et al., 2013). An earlier study reported that HCA caused reduction of food intake and body weight gain in rats given high sucrose, high glucose, and high sucrose with fat (Leonhardt et al., 2001). HCA was found to inhibit the function of ATP-citrate lyase by preventing the conversion of citrate into oxaloacetate and acetyl-CoA (Amran et al., 2009). Limited acetyl-CoA perturbs the biosynthesis of fatty acid and cholesterol. Inhibition of acetyl-CoA production directly depletes the amount of subsequent metabolites (called malonyl-CoA) in the fatty acid synthetic pathway. Malonyl inhibits the enzyme carnitine acyltransferase from oxidizing fat. Indirectly, it is proposed that HCA reduces the formation of malonyl-CoA and therefore stimulates fat metabolism. Due to these metabolic alterations, a signal is then delivered to the brain to increase the serotonin level with a concomitant reduction in appetite. In human trials, HCA has been reported to cause a profound reduction in appetite and weight loss concomitant with an improved lipid profile (Chuah et al., 2013).

In this study, treatments with MeGa and Adipex reduced the levels of total cholesterol, triglyceride, and LDL in the obese rats. This suggests that MeGa possesses antiobesity activity with an improvement of lipid profiles, as displayed by the obese rats. As previously mentioned, HCA is a competitive inhibitor of ATP-citrate lyase, which controls the availability of acetyl-CoA for the synthesis of cholesterol and triglyceride. HCA has also been found to reduce cholesterol levels through a series of processes, such as interruption of intestinal cholesterol uptake, conversion of cholesterol into bile acids, and excretion of intestinal bile acids. HCA decreases the levels of cholesterol and triglyceride by inhibiting the endogenous synthesis of cholesterol and triglyceride via inhibition of the synthesis of acetyl-CoA.

The score plot from principal component analysis (PCA) illustrated an obvious separation between the lean rats and other groups of rats before and after treatment regimes. Metabolic pathway analysis (MetPA) revealed 22 affected metabolic pathways which differentiated MeGa-treated obese rats from lean rats. These pathways include the biosynthesis of unsaturated fatty acids and steroid, metabolism of tyrosine, retinol, tryptophan, sphingolipid, and alpha-linolenic acid. Comparing the metabolome of these 4 studied groups (lean, obese, Adipex-treated, Ga-2-treated), most of the identified metabolites and metabolism pathways were linked to the control of energy balance and the role of mitochondria. Metabolites linked with these metabolism pathways are acylcarnitine, phosphatidylcholine, L-kyeunerine, and arachidonic acid. Acylcarnitine is the main transporter involved in the energy production process. It carries activated long-chain fatty acids (the breakdown products of fatty acids and amino acids) into mitochondria for the β-oxidation process (Tarasenko et al., 2018). Acylcarnitine also serves as an important biomarker of mitochondrial dysfunction with an increase of acylcarnitines (Chen et al., 2015).

Lipid is an important source of energy reserves. Elevated lipid profiles (increase fatty acids or cholesterol) in the bloodstream are associated with an increased risk for cardiovascular-related diseases, including coronary heart disease (excessive fat deposition in blood vessels), diabetes (high blood sugar), and hypertension (high blood pressure). In the hepatic and muscle mitochondria, free fatty acids (FFAs) are broken down to generate energy through catabolic processes such as the fatty acid β-oxidation and tricarboxylic acid cycle (TCA cycle). Excess lipids are stored in fat tissues (adipose tissue) and accumulated elsewhere in the form of triglycerides. Obesity is characterized by increased lipid deposition and decreased lipid removal in tissues, as well as with an elevated level of FFAs in plasma. In line with this, the alteration of serum FFA levels in obese animals was investigated using metabolomics analysis to decipher the affected pathways. Furthermore, saturated fatty acids (SFAs) in the composition of serum FFAs have been linked to the development of obesity.

In the metabolomics analysis, the highest impact score is that of the biosynthesis of fatty acids. Malonyl-CoA not only serves as an essential substrate for the biosynthesis of fatty acids, but also limits the supply of fatty acid into mitochondria. In other words, the availability of fatty acids in mitochondria stimulates the lipogenesis process and directly accelerates oxidation of glucose in response to acetyl-CoA carboxylase, which is activated by insulin. A previous study had reported that obese individuals have higher malonyl-CoA levels but lower rates of fatty acid oxidation compared to healthy individuals (Chen et al., 2015). Apart from malonyl-CoA, other compounds detected were acetyl carnitine and phosphatidylcholine. Acetyl carnitine is involved in the metabolism of fatty acids; it is one of the important metabolites associated with obesity. Fatty acids undergo oxidation actively with the provision of FFAs in the body. In the presence of acetyl carnitine, fatty acids undergo esterification and are transferred to mitochondria for subsequent β-oxidation to produce energy. Phosphatidylcholine is the major phospholipid component found in all plasma lipoprotein. In the liver, biosynthesis of phosphatidylcholine is important for the secretion of plasma lipoproteins such as very low density lipoprotein (VLDL) and high density lipoprotein (HDL). In the cell membranes, lipolytic enzymes (e.g., phospholipase D) metabolize phosphatidylcholine into several isoforms of lipids (e.g., phosphatidic acid and choline). Hepatic and serum phosphatidylcholine levels were higher in obese mice than in normal mice (Kim et al., 2011). Choline, an essential dietary nutrient, not only protects the integrity of cellular structure, but also regulates one-carbon metabolism (methyl, CH3) and transportation of lipid/cholesterol. In animals, the synthesis of phosphatidylcholine via the Kennedy pathway requires a high percentage of choline (more than 95%) in the tissues (Xie et al., 2012). For the synthesis of methionine from homocysteine, choline oxidizes to betaine in the mitochondria of the liver and kidney to supply the methyl groups.

Apart from that, the branched chain amino acids (BCAAs), which are nutritionally essential for the metabolism of amino acids, were detected in this study. They are N-acetylserotonin, 4, 6-dihydroxyquinoline, L-kynurenine, L-normetanephrine, 4-hydroxyphenylacetaldehyde, 4-fumarylacetoacetate, and S-methyl-5-thio-D-ribose 1-phosphate. The BCAAs are important in regulating synthesis of protein, metabolism of glucose, and oxidation, as well as secretion of leptin from fat (Xie et al., 2012). These BCAAs as well as aromatic amino acids might be used to promote synthesis of protein by changing the metabolic direction of amino acids, as indicated in HCA-treated male rats (Han et al., 2016a). Therefore, it is postulated that obesity may perturb the catabolism pathways of BCAA.

HCA is structurally similar to citrate, which plays an important role in allosteric regulation for a number of enzymes involved in the metabolism of carbohydrates and fats (Han et al., 2016a). The metabolomics approach adopted by our study further supported that the pathways involved in the metabolism of lipid (glycerophospholipid) and biosynthesis of unsaturated fatty acid were perturbed in rats treated with MeGa. Similar to another study by Liu et al. (2015), we also observed changes in the lipid profiles of the rats treated with MeGa. The mechanisms associated with such findings are explained by the inhibition of ATP-citrate lyase (ACLY) activity. In addition, Liu et al. (2015) further explained that the reduced fat accumulation in the liver of HFD rats treated with *Garcinia cambogia* mainly occurred due to the lipolysis of exogenous triglyceride, as observed with the increase of the mRNA levels of adipose triglyceride lipase. In addition, the adiponectin–AMPK signalling pathway was also stimulated with increased mRNA levels of adenosine 5’-monophosphate-activated protein kinase (AMPK) α1 and adiponectin receptor 1. However, there was no suppression of the synthesis of endogenous triglyceride, as no change to the mRNA levels of ACLY, fatty acid synthase (FAS), or Acyl-CoA oxidase genes were found (Liu et al., 2015). However, (-)-HCA-treated broiler chicken and primary culture of chicken hepatocytes have been reported to show changes in the hepatic mRNA expressions involved in the metabolism of lipids. Expression of genes related to the synthesis of fatty acids such as FAS, sterol regulatory element binding protein-1c (SREBP-1c)], and ACLY genes were downregulated, while the peroxisome proliferators-activated receptor α (PPARα) gene was upregulated (Han et al., 2016b; Li et al., 2017). Further metabolomics investigation indicated that (-)-HCA was involved in the metabolisms of amino acids, protein synthesis, citric acid cycle, and synthesis of uric acid and fatty acids in controlling weight gain and lipid accumulation (Peng et al., 2017). Our findings thus far support that MeGA reduced body weight and maintained desirable lipid profiles via its control over the metabolism of lipids which might be regulated by the differential expression of genes, such as FAS, SREBP-1c, ACLY, and PPARα. However, further investigations into their precise mechanisms are required.

Current research findings have demonstrated that MeGa successfully reduced body weight gain and maintained desirable lipid profiles in the obese rats. It is a good weight management alternative and safe for consumption according to the safety information obtained from the OECD guidelines. There are two (2) main benefits observed in rats treated with MeGa: (i) reduced weight gain by reduction in the amount of food intake (suppressed food intake), and (ii) improved lipid profiles in the alteration of the biosynthesis of fatty acids and metabolism of lipids. Therefore, MeGA is a safer alternative that can be consumed as part of a weight management programme.

Supplementary Materials

## References

[ref1] (2018). Combined extract of Moringa oleifera and Centellaasiatica modulates oxidative stress and senescence in hydrogen peroxide-induced human dermal fibroblasts. Turkish Journal of Biology.

[ref2] (2013). Study on the relationship of the phenolic, flavonoid and tannin content to the antioxidant activity of Garcinia atroviridis. Universal Journal of Applied Science.

[ref3] (2015). Metabolomics - the complementary field in systems biology: a review on obesity and type 2 diabetes. Molecular BioSystems.

[ref4] (2017). Anti-obesity potential of selected tropical plants via pancreatic lipase inhibition. Advances in Obesity Weight Management & Control6.

[ref5] (2014). Review on some Malaysian traditional medicinal plants with therapeutic properties. Journal of Basic and Applied Sciences.

[ref6] (2009). Effects of Garcinia atroviridis on serum profiles and atherosclerotic lesions in the aorta of guinea pigs fed a high cholesterol diet. Singapore Medical Journal.

[ref7] (2014). Bioactive components and antioxidant properties of Terminalia arjuna L. extracts. Journal of Food Processing & Technology.

[ref8] (2015). Chang C-S et al. International Journal of Obesity.

[ref9] (2013). Updates on antiobesity effect of Garcinia origin (-)-. HCA. Evidence-Based Complementary and Alternative Medicine.

[ref10] (2012). In vitro and in vivo toxicity of Garcinia or hydroxycitric acid: A review. Evidence-Based Complementary and Alternative Medicine.

[ref11] (2019). Profound perturbation of the metabolome in obesity is associated with health risk. Cell Metabolism.

[ref12] (2018). Acute liver injury following Garcinia cambogia weight-loss supplementation: case series and literature review. Internal and Emergency Medicine13.

[ref13] (2017). Garcinia atroviridis – A review on phytochemicals and pharmacological properties. Marmara Pharmaceutical Journal.

[ref14] -Hydroxycitric acid nourishes protein synthesis via altering metabolic directions of amino acids in male rats. Phytotherapy Research.

[ref15] (2016b). -Hydroxycitric acid reduced fat deposition via regulating lipid metabolism-related gene expression in broiler chickens. Lipids in Health and Disease.

[ref16] (1998). Phytochemical methods: a guide to modern techniques of plant analysis.

[ref17] (2009). The effect of eating behavior on weight loss and maintenance during a lifestyle intervention. Preventive Medicine.

[ref18] (2011). Metabolomic analysis of livers and serum from high-fat diet induced obese mice. Journal of Proteome Research.

[ref19] (2005).

[ref20] (2018). Hepatotoxicity associated with use of the weight loss supplement Garcinia cambogia: a case report and review of the literature. Case Reports in Hepatology.

[ref21] (2001). Effect of hydroxycitrate on food intake and body weight regain after a period of restrictive feeding in male rats. Physiology & Behavior74.

[ref22] (2017). -Hydroxycitric acid reduced lipid droplets accumulation via decreasing acetyl-Coa supply and accelerating energy metabolism in cultured primary chicken hepatocytes. Cellular Physiology and Biochemistry.

[ref23] (2015). Garcinia Cambogia extracts prevented fat accumulation via adiponectin-AMPK signaling pathway in developing obesity rats. Food Science and Technology Research.

[ref24] (2017). Benefits of AsamGelugur (Garcinia atroviridis) tea as a source of antioxidant compounds on malondialdehyde levels in adults with obesity. American Scientific Research Journal of Engineering Technology and Sciences.

[ref25] (2000). Antimicrobial, antioxidant, antitumour-promoting and cytotoxic activities of different plant part extracts of Garcinia atroviridis Griff. Journal of Ethnopharmacology.

[ref26] (2012). Nutritional content and in vitro antioxidant potential of Garcinia atroviridis (asamgelugor) leaves and fruits. Malaysian Journal of Nutrition.

[ref27] (2009). Facultative apomixis in Garcinia atroviridis (clusiaceae) and effects of different pollination regimes on reproductive success. Tropical Life Sciences Research.

[ref28] (2015). A metabolomic approach to understand the metabolic link between obesity and diabetes. Molecules and Cells.

[ref29] (2017). Metabolomics reveals the mechanism of (-)-hydroxycitric acid promotion of protein synthesis and inhibition of fatty acid synthesis in broiler chickens. Animal.

[ref30] (2006). Drying Characteristics of Garcinia atroviridis. Walailak Journal of Science and Technology.

[ref31] (2014). Weight loss maintenance: a review on dietary related strategies. Journal of Research in Medical Sciences.

[ref32] (2018). Tissue acylcarnitine status in a mouse model of mitochondrial beta-oxidation deficiency during metabolic decompensation due to influenza virus infection. Molecular Genetics and Metabolism.

[ref33] (2013). Animal models of dietary-induced obesity. Animal Models for the Study of Human Disease.

[ref34] (2012). Investigating potential mechanisms of obesity by metabolomics. Journal of Biomedicine and Biotechnology.

[ref35] (2011). Antioxidant compounds from propolis collected in Anhui, China. Molecules.

[ref36] (2002). Utilization of some Garcinia species in Thailand. Acta Horticulturae.

