# Supplementary material for: The methanolic extract of Garcinia atroviridis (MeGa) reduces body weight and food intake, and improves lipid profiles by altering the lipid metabolism: a rat model
Source: Turk J Biol. 2020 Dec 14;44(6):437–48. doi: 10.3906/biy-2005-2 (PMC7759190; doi:10.3906/biy-2005-2)
Supplement: Supplementary file 1 — Supplementary Materials [file turkjbio-44-437-sup001.pdf]

Supplementary data

Table A1. Ingredient compositions of experimental diets according to the acute, subacute toxicity and antiobesity studies. Abbreviation: Ctrl = control; Sat. = satellite.

Class	Ingredients / Caloric information	Acute toxicity study		Subacute toxicity study			Antiobesity study			
		Ctrl	Test	Ctrl	Test	Sat.	Lean	Obese	Adipex-treated obese	MeGa-treated obese
		NFD	NFD	NFD	NFD	NFD	NFD	HFD	HFD	HFD
Protein	Casein, Lactic, 30 Mesh	189.56	189.56	189.56	189.56	189.56	189.56	258.45	258.45	258.45
	L-Cystine	2.84	2.84	2.84	2.84	2.84	2.84	3.88	3.88	3.88
Carbohydrate	Sucrose, Fine granulated	335.53	335.53	335.53	335.53	335.53	335.53	94.08	94.08	94.08
	Corn starch	298.56	298.56	298.56	298.56	298.56	298.56	-	-	-
	Lodex 10	33.17	33.17	33.17	33.17	33.17	33.17	161.53	161.53	161.53
Fiber	Solka Floc, FCC200	47.39	47.39	47.39	47.39	47.39	47.39	64.61	64.61	64.61
Fat	Soybean oil	23.70	23.70	23.70	23.70	23.70	23.70	32.31	32.31	32.31
	Lard	18.96	18.96	18.96	18.96	18.96	18.96	316.60	316.60	316.60
Mineral	Mineral mix	47.39	47.39	47.39	47.39	47.39	47.39	64.61	64.61	64.61
Vitamin	Choline Bitartrate	1.90	1.90	1.90	1.90	1.90	1.90	2.58	2.58	2.58
	Vitamin mix	0.95	0.95	0.95	0.95	0.95	0.95	1.29	1.29	1.29
Dye	Yellow dye (FD&C #5)	0.05	0.05	0.05	0.05	0.05	0.05	-	-	-
	Blue dye (FD&C #1)	-	-	-	-	-	-	0.06	0.06	0.06
	Total (g)	1000	1000	1000	1000	1000	1000	1000	1000	1000
Caloric information	Protein (% Kcal)	20	20	20	20	20	20	20	20	20
	Fat (% Kcal)	10	10	10	10	10	10	60	60	60
	Carbohydrate (% Kcal)	70	70	70	70	70	70	20	20	20
	Energy density (Kcal/g)	3.82	3.82	3.82	3.82	3.82	3.82	5.21	5.21	5.21
Oral administration		Distilled water	2000 mg/kg MeGa	Distilled water	1000 mg/kg MeGa	1000 mg/kg MeGa	Distilled water	Distilled water	15 mg/kg Adipex	100 mg/kg (Ga-1), 200 mg/kg (Ga-2), 400 mg/kg (Ga-3)

Normal fat diet (NFD), also called chow diet (D12450B, Research Diets, USA); high-fat diet (HFD) (D12492, Research Diets, USA); Methanolic extract of *Garcinia atroviridis* (MeGa) was used for toxicity study (acute and subacute) and antiobesity study. There are two groups in acute toxicity study: control (chow diet) and test (chow diet) + 2000 mg/kg MeGa groups. There are three groups in subacute toxicity study: control (chow diet), test (chow diet) + 1000 mg/kg MeGa and satellite (chow diet) + 1000 mg/kg MeGa groups. For antiobesity study, there are four main groups: lean (NFD), obese (HFD), Adipex-treated obese (HFD + 15 mg/kg Adipex) and three subgroups of MeGa-trated obese [Ga-1:(HFD + 100 mg/kg), Ga-2:(HFD + 200 mg/kg), Ga-3:(HFD + 400 mg/kg)].

Table A2. Metabolite profiling of MeGa using LCMS – QTOF in positive ESI mode.

Compound name	Retention time	Molecular Formula	Molecular weight	Compound structure
Tartaric acid	0.842	$C_4H_6O_6$	150.0278	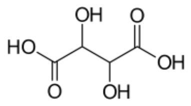
Ascorbic acid	2.343	$C_6H_8O_6$	176.0325	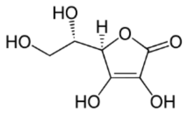
Hydroxycitric acid	14.608	$C_6H_8O_8$	208.1095	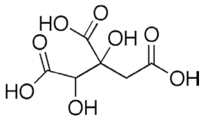
2-(butoxycarbonylmethyl)-3-butoxycarbonyl-2-hydroxy-3-propanolide	22.87	$C_{14}H_{23}O_7$	303.2912	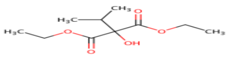
Malic acid	23.55	$C_4H_6O_5$	134.1091	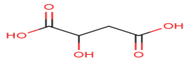

Table A3. Subacute toxicity effects of MeGa extract for 28 days. (a) Body weight, (b) food intake, (c) haematological analysis, (d) biochemical analysis (liver and kidney function test) and (e) relative organ weight in male and female rats for normal control, test (treated with 1000 mg/kg MeGa) and satellite group (treated with 1000 mg/kg MeGa). Values are in mean ± SEM.

Parameters (Unit)	Normal control		Test		Satellite	
	Male	Female	Male	Female	Male	Female
(a) Body weight (g)	420.90 ± 13.66	256.70 ± 4.34	370.50 ± 8.27	247.10 ± 7.16	422.11 ± 8.20	248.00 ± 4.65
(b) Food intake (g)	85.36 ± 4.50	61.35 ± 0.18	77.10 ± 2.53	58.43 ± 2.26	84.93 ± 2.31	57.66 ± 2.74
(c) Haematological analysis						
White blood cell count (10^9/L)	6.53 ± 0.80	6.69 ± 1.24	8.79 ± 1.86	6.53 ± 2.33	9.53 ± 1.97	9.33 ± 1.79
Neutrophils (10^9/L)	0.20 ± 0.39	0.33 ± 0.15	0.16 ± 0.01	0.48 ± 0.14	0.28 ± 0.04	0.34 ± 0.14
Lymphocytes (10^9/L)	3.12 ± 0.71	3.64 ± 1.06	4.40 ± 1.49	5.93 ± 1.21	5.02 ± 1.49	6.28 ± 1.44
Monocytes (10^9/L)	1.20 ± 0.30	1.58 ± 0.29	1.60 ± 0.09	2.07 ± 0.43	2.27 ± 0.28	1.54 ± 0.22
Eosinophils (10^9/L)	0.09 ± 0.01	0.16 ± 0.02	0.11 ± 0.02	0.12 ± 0.02	0.17 ± 0.03	0.13 ± 0.04
Red blood cell count (10^{12}/L)	8.33 ± 0.09	8.36 ± 0.15	8.77 ± 0.27	8.15 ± 0.11	8.34 ± 0.11	8.43 ± 0.08
Hemoglobin (g/dL)	15.03 ± 0.18	16 ± 0.29	16.38 ± 0.55	15.2 ± 0.38	15.95 ± 0.20	15.83 ± 0.44
Hematocrit (%)	46.58 ± 1.52	47.17 ± 1.21	50.17 ± 1.05	47.2 ± 0.61	47.40 ± 0.33	45.43 ± 0.72
Mean red blood cell volume (MCV) (fL)	55.95 ± 1.57	56.58 ± 0.89	58.23 ± 0.52	55.63 ± 0.64	55.50 ± 1.24	54.10 ± 0.50
Mean corpuscular Hb (pg)	18.08 ± 0.24	18.90 ± 0.06	18.66 ± 0.18	18.88 ± 0.33	19.11 ± 0.28	18.50 ± 0.40
Mean corpuscular Hb concentration (MCHC) (g/dL)	32.30 ±0.76	32.63 ± 0.47	32.42 ± 0.31	32.93 ± 0.34	33.80 ± 0.32	34.65 ± 0.65
(d) Biochemical analysis						
ALP (U/L)	135.8 ± 5.05	72.18 ± 6.24	127.52 ± 7.95	88.27 ± 6.24	121.70 ± 5.00	66.87 ± 4.26
ALT (U/L)	45.52 ± 2.12	45.75 ± 3.02	47.53 ± 2.76	43.62 ± 3.82	43.58 ± 1.96	42.32 ± 2.02
AST (U/L)	125.07 ± 7.77	143.58 ± 11.85	161.48 ± 10.58	148.97 ± 6.53	122.07 ± 5.09	152.40 ± 14.9
CRE (μmol/L)	29.67 ± 2.20	39.50 ± 1.43	34.33 ± 2.51	41.33 ± 3.11	30.67 ± 1.69	36.50 ± 2.77
(e) Relative organ weight						
Liver (%)	2.76 ± 0.05	3.02 ± 0.08	2.88 ± 0.06	3.16 ± 0.06	2.84 ± 0.12	2.92 ± 0.09
Heart (%)	0.35 ± 0.02	0.39 ± 0.01	0.39 ± 0.02	0.40 ± 0.01	0.34 ± 0.02	0.36 ± 0.02
Lung (%)	0.56 ± 0.05	0.67 ± 0.02	0.53 ± 0.01	0.76 ± 0.03	0.47 ± 0.01	0.68 ± 0.03
Kidney (%)	0.70 ± 0.01	0.72 ± 0.02	0.75 ± 0.03	0.75 ± 0.01	0.67 ± 0.02	0.74 ± 0.02
Spleen (%)	0.16 ± 0.01	0.19 ± 0.01	0.17 ± 0.01	0.21 ± 0.01	0.19 ± 0.01	0.18 ± 0.01
Brain (%)	0.51 ± 0.01	0.74 ± 0.02	0.54 ± 0.01	0.78 ± 0.03	0.49 ± 0.01	0.77 ± 0.01

Table A4. List of significantly differentiated metabolites and metabolism pathways in different treatment groups. (a) Lean versus obese, (b) lean versus Adipex and (c) lean versus MeGa (200 mg/kg). Abbreviation: n.d = not detected, dw = down.

Pathway	Metabolism	Metabolites	Mass	(a) Lean versus obese		(b) Lean versus Adipex		(c) Lean versus MeGa	
				pre	post	pre	post	pre	post
Lipid metabolism	Biosynthesis of unsaturated fatty acids	7Z, 10Z, 13Z, 16Z, 19Z-docosapentaenoic acid	330.2568	n.d	up	n.d	up	n.d	up
		4Z,7Z,10Z,13Z,16Z,19Z)-4,7,10,13,16,19-Docosahexaenoic acid	256.2392	n.d	dw	n.d	up	n.d	up
		5,8,11,14,17-Icosapentaenoic acid	302.2237	dw	dw	dw	up	dw	up
		Linoleic acid	280.2398	n.d	up	n.d	dw	n.d	dw
		Arachidonic Acid (peroxide free)	248.0539	n.d	dw	n.d	up	n.d	up
		α-Linolenic Acid	278.2241	up	up	up	up	up	up
	Sphingolipid metabolism	Sphinganine	301.2977	n.d	up	n.d	up	n.d	up
		Sphingosine-1-phosphate	379.2496	n.d	up	n.d	dw	n.d	dw
		Sphinganine-phosphate	381.2635	n.d	up	n.d	dw	n.d	dw
		3-ketosphinganine	299.2849	dw	up	dw	up	dw	up
	Steroid hormone biosynthesis	21-Hydroxypregnenolone	348.2299	n.d	dw	n.d	up	n.d	up
		progesterone	314.2246	n.d	dw	n.d	dw	n.d	dw
		17a-Hydroxyprogesterone	330.2191	n.d	dw	n.d	up	n.d	up
		17alpha,21-Dihydroxypregnenolone	348.2289	n.d	up	n.d	up	n.d	up
		(20S)-17,20-dihydroxypregn-4-en-3-one	232.1459	n.d	up	n.d	up	n.d	up
		Corticosterone	346.2148	n.d	dw	n.d	up	n.d	up
		11-Dehydrocorticosterone	344.1987	n.d	up	n.d	up	n.d	up
	Steroid biosynthesis	Calcidiol	400.3359	n.d	dw	n.d	up	n.d	up
		Calcitriol	410.3525	n.d	up	n.d	up	n.d	up
		Campesterol	400.3722	n.d	up	n.d	up	n.d	up
		4,4-Dimethylcholesta-8,14,24-trienol	416.328	n.d	up	n.d	up	n.d	up
	Primary bile acid biosynthesis	3alpha,7alpha,26-Trihydroxy-5beta-cholestane	420.3606	n.d	dw	n.d	up	n.d	up
		Cholest-5-ene-3beta,26-diol	402.3506	n.d	dw	n.d	dw	n.d	dw
		3α,7α-Dihydroxy-5β-cholestanate	434.34	n.d	dw	n.d	up	n.d	up
	Glycerophospholipid metabolism	Phosphatidylethanolamine	773.5383	n.d	up	n.d	up	n.d	up
		Phosphatidylcholine	805.5603	dw	up	dw	up	dw	up
Metabolism of cofactors and vitamins	Retinol metabolism	9-cis-retinal	284.2134	up	up	up	up	up	up
		9-cis-Retinoic acid	300.208	up	up	up	dw	up	dw
		11-cis-Retinol	286.2287	n.d	up	n.d	dw	n.d	dw
Signalling molecules and interaction	Neuroactive ligand-receptor interaction	Anandamide	328.2611	n.d	dw	n.d	dw	n.d	dw
		Arachidonoyl dopamine	439.3095	n.d	up	n.d	dw	n.d	dw
Amino acid metabolism	Tryptophan metabolism	N-Acetylserotonin	218.1049	n.d	up	n.d	up	n.d	up
		4,6-Dihydroxyquinoline	161.0463	n.d	dw	n.d	up	n.d	up
		L-Kynurenine	208.0836	n.d	up	n.d	up	n.d	up
	Tyrosine metabolism	L-Normetanephrine	183.0902	n.d	up	n.d	dw	n.d	dw
		4-Hydroxyphenylacetaldehyde	136.0527	n.d	up	n.d	up	n.d	up
		4-Fumarylacetoacetate	200.0339	n.d	up	n.d	up	n.d	up
	Cysteine and methionine metabolism	S-Methyl-5-thio-D-ribose 1-phosphate	260.0095	n.d	dw	n.d	up	n.d	up

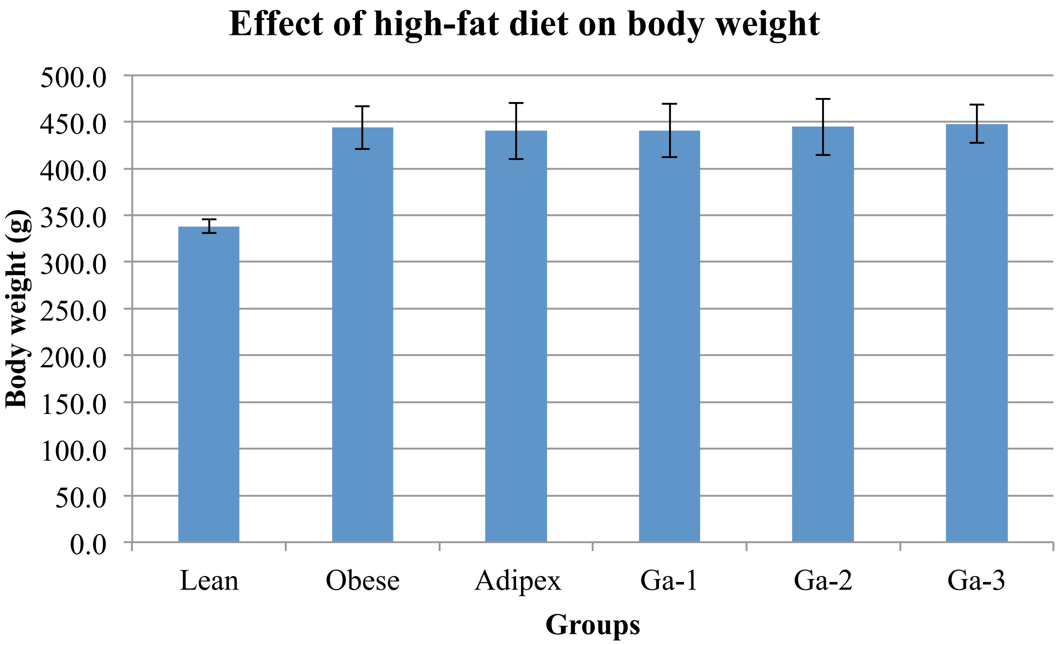

Figure A1. Effect of high-fat diet on body weight of the pretreatment lean group and moderately obese groups, including obese, Ga-1 (100 mg/kg MeGa), Ga-2 (200 mg/kg MeGa) and Ga-3 (400 mg/kg MeGa) groups.

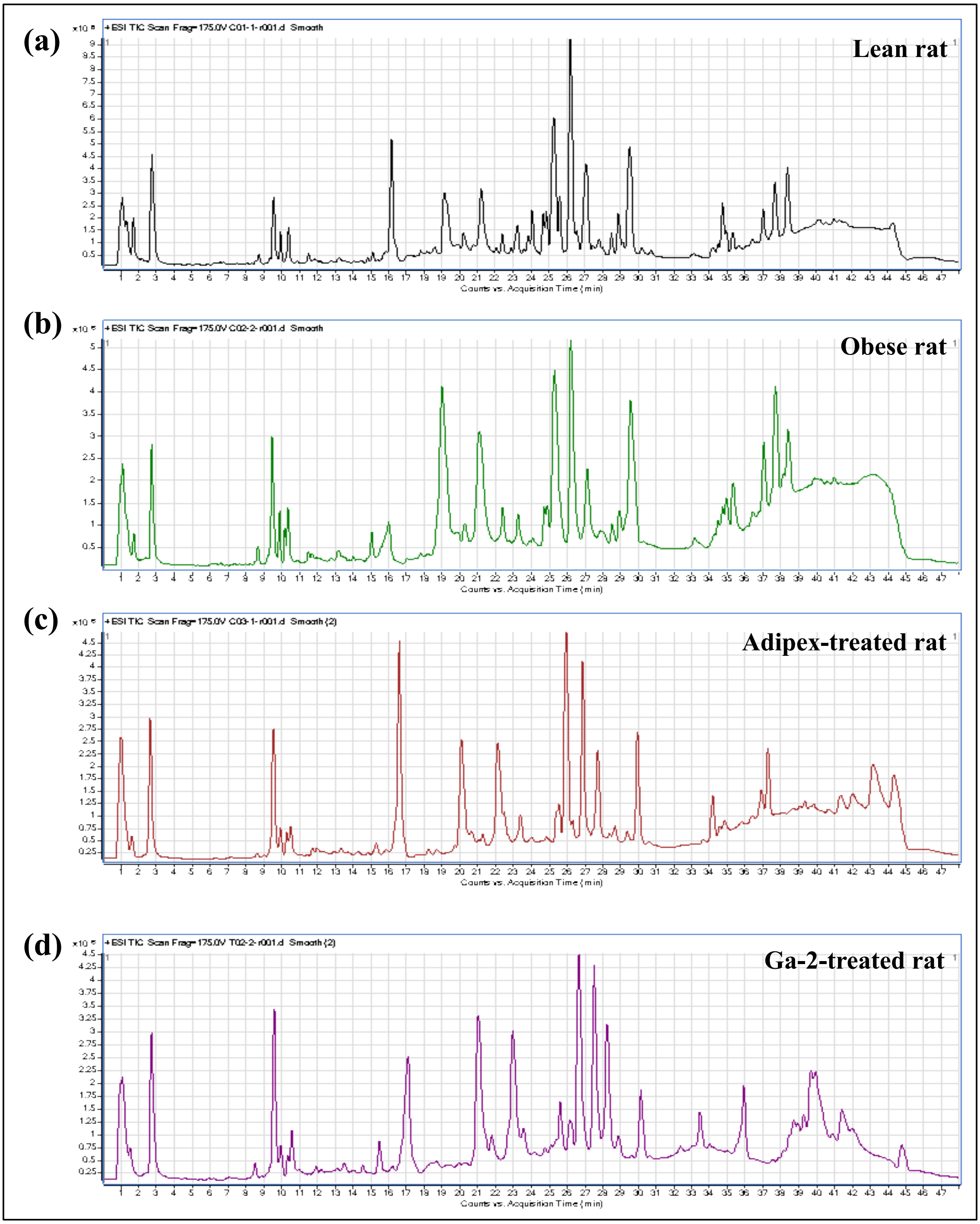

Figure A2. LC/MS Q-TOF chromatography of serum samples. (a) Lean rat, (b) obese rat, (c) Adipex-treated rat, and (d) MeGa-treated rat.
